# *In vivo* Imaging With ^18^F-FDG- and ^18^F-Florbetaben-PET/MRI Detects Pathological Changes in the Brain of the Commonly Used 5XFAD Mouse Model of Alzheimer's Disease

**DOI:** 10.3389/fmed.2020.00529

**Published:** 2020-09-15

**Authors:** Timon N. Franke, Caroline Irwin, Thomas A. Bayer, Winfried Brenner, Nicola Beindorff, Caroline Bouter, Yvonne Bouter

**Affiliations:** ^1^Department of Psychiatry and Psychotherapy, University Medical Center Göttingen (UMG), Georg-August-University, Göttingen, Germany; ^2^Department of Nuclear Medicine, Charité-Universitätsmedizin Berlin, Berlin, Germany; ^3^Berlin Experimental Radionuclide Imaging Center (BERIC), Charité-Universitätsmedizin Berlin, Berlin, Germany; ^4^Department of Nuclear Medicine, University Medical Center Göttingen (UMG), Georg-August-University, Göttingen, Germany

**Keywords:** FDG-PET, Amyloid-PET, Florbetaben, Alzheimer's disease, 5XFAD Alzheimer model

## Abstract

Imaging biomarkers of Alzheimer's disease (AD) that are able to detect molecular changes *in vivo* and transgenic animal models mimicking AD pathologies are essential for the evaluation of new therapeutic strategies. Positron-emission tomography (PET) using either ^18^F-Fluorodeoxyglucose (^18^F-FDG) or amyloid-tracers is a well-established, non-invasive tool in the clinical diagnostics of AD assessing two major pathological hallmarks. ^18^F-FDG-PET is able to detect early changes in cerebral glucose metabolism and amyloid-PET shows cerebral amyloid load. However, the suitability of ^18^F-FDG- and amyloid-PET in the widely used 5XFAD mouse model of AD is unclear as only a few studies on the use of PET biomarkers are available showing some conflicting results. The aim of this study was the evaluation of ^18^F-FDG-PET and amyloid-PET in 5XFAD mice in comparison to neurological deficits and neuropathological changes. Seven- and 12-month-old male 5XFAD mice showed a significant reduction in brain glucose metabolism in ^18^F-FDG-PET and amyloid-PET with ^18^F-Florbetaben demonstrated an increased cerebral amyloid deposition (*n* = 4–6 per group). Deficits in spatial reference memory were detected in 12-month-old 5XFAD mice in the Morris Water Maze (*n* = 10–12 per group). Furthermore, an increased plaque load and gliosis could be proven immunohistochemically in 5XFAD mice (*n* = 4–6 per group). PET biomarkers ^18^F-FDG and ^18^F-Florbetaben detected cerebral hypometabolism and increased plaque load even before the onset of severe memory deficits. Therefore, the 5XFAD mouse model of AD is well-suited for *in vivo* monitoring of AD pathologies and longitudinal testing of new therapeutic approaches.

## Introduction

Alzheimer's disease (AD) is the most common form of dementia with an estimated number of more than 40 Million cases worldwide (1). Due to the demographic trend of an aging population, an increasing incidence of the disease is anticipated, while there is no cure available so far. Major pathological hallmarks of AD include the formation of extracellular beta-amyloid (Aβ) plaques, neurofibrillary tangles, neuroinflammation, and neuron loss (2). To date, a final diagnosis of the disease can only be made by histopathological detection of amyloid plaques and neurofibrillary tangles *post mortem*.

However, in order to establish a probable diagnosis of AD, biomarkers that are able to detect pathological hallmarks of the disease *in vivo*, are essential. Next to the measurement of Aβ42 as well as total tau and phosphorylated tau in cerebrospinal fluid, clinical biomarkers also include magnetic resonance imaging (MRI) and molecular imaging with positron-emission tomography (PET) (3–5).

PET represents a non-invasive imaging tool for the detection of AD hallmarks. Two major pathological hallmarks of AD can be assessed with the clinically established imaging biomarkers ^18^F-Fluorodeoxyglucose- (FDG) and amyloid-PET in order to determine synaptic dysfunction and cerebral amyloid load, respectively (3).

In order to study pathomechanisms and develop novel treatment strategies for AD, preclinical models of the disease are crucial. A preclinical model of AD should reveal pathological features of the disease as accurately as possible. Next to modeling pathological hallmarks, features of a valid model also should include imaging of these pathologies *in vivo* using the same biomarkers as used in clinical routine.

A commonly used and well-established mouse model in AD research is the 5XFAD model of brain amyloidosis that carries five familial human AD mutations in the amyloid precursor protein (APP) and the presenilin-1 (PSEN1) gene (6). 5XFAD mice recapitulate many AD-related phenotypes and develop an aggressive and progressive plaque pathology as well as synaptic dysfunctions and neuron loss. In addition, 5XFAD mice show age-dependent behavioral deficits (7–10). While 5XFAD mice display features of AD that can be assessed by FDG- and amyloid-PET, only a few studies using PET in 5XFAD mice are available so far, with partly contradictory findings (11–13).

The aim of this study was the evaluation of the clinically established AD-biomarkers ^18^F-FDG-and ^18^F-Florbetaben-PET in the commonly used 5XFAD mouse model in order to assess whether PET can be used as a reliable tool for longitudinal disease assessment, and thus for therapy response monitoring in the future.

## Materials and Methods

### 5XFAD Transgenic Mice

The 5XFAD mouse model of cerebral amyloidosis (Jackson Laboratories, Bar Harbor, ME, United States) was first described by Oakley et al. (6). In short, this double transgenic mouse model carries five mutations found in patients with familial AD. The 695 amino acids isoform of the human amyloid precursor protein (APP695) is over-expressed carrying the Swedish (K670N/M671L), Florida (I716V), and London (V717I) mutations under the control of the murine Thy-1 promoter. In addition, human presenilin-1 (PSEN-1) carrying the M146L and L286V mutations are also expressed under the control of the murine Thy-1 promoter. 5XFAD mice used in this study were kept on a C57Bl/6J genetic background and wild type littermates served as age-matched control animals.

All animals were handled in accordance with the German guidelines for animal care. All experiments were approved by the local authorities (Niedersächsisches Landesamt für Verbraucherschutz und Lebensmittelsicherheit, Röverskamp 5, 26203 Oldenburg, Germany and Landesamt für Gesundheit und Soziales LAGeSo Darwinstr. 15, 10589 Berlin, Germany).

### ^18^F-FDG-PET/MRI

^18^F-FDG-PET/MRI was performed on 7- and 12-month-old male 5XFAD mice as well as age- and sex-matched C57Bl/6J wild type mice (*n* = 4–6 per group). Mice were fasted overnight and blood glucose levels were measured in a blood sample from a tail vein before tracer injection. Blood glucose levels were 89–248 mg/dl (mean 158 mg/dl) while there were no significant differences between groups. ^18^F-FDG (11.46–20.53 MBq; mean 16.81 MBq) was injected into a tail vein with a maximum volume of 200 μl followed by an uptake period of 45 min. Mice were awake during the uptake process. PET scans were performed for 20 min using a small animal 1 Tesla nanoScan PET/MRI (Mediso, Hungary). Mice were anesthetized with isoflurane supplemented with oxygen during the scans and kept on a heated bed (37°C). Respiratory rate was measured throughout the imaging process. MRI-based attenuation correction was conducted with the material map (matrix 144 × 144 × 163 with a voxel size of 0.5 × 0.5 × 0.6 mm^3^, repetition time: 15 ms, echo time: 2.032 ms and a flip angle of 25°) and the PET images were reconstructed using the following parameters: matrix 136 × 131 × 315, voxel size 0.23 × 0.3 × 0.3 mm3.

### ^18^F-Florbetaben-PET/MRI

^18^F-FBB-PET/MRI was performed on 7- and 12-month-old male 5XFAD mice as well as age- and sex-matched C57Bl/6J wild type mice after ^18^F-FDG-PET imaging (*n* = 4–6 per group). In isoflurane anesthetized mice ^18^F-Florbetaben (7.5–24 MBq; mean 14 MBq) was administered intravenously with a maximum volume of 200 μl (14). PET acquisition of 30 min duration started after an uptake period of 40 min. Animals remained in anesthesia during the uptake period. MRI-based attenuation correction was conducted with the material map, and the PET images were reconstructed as described for ^18^F-FDG-PET/MRI.

### Image Analysis

All images were analyzed using PMOD v3.9 (PMOD Technologies, Switzerland) as previously described (15). Briefly, a predefined MRI-based mouse brain atlas template was used to define different volumes of interest (VOI) including whole brain volume as well as the amygdala, brain stem, cerebellum, cortex, hippocampus, hypothalamus, midbrain, olfactory bulb, septum/basal forebrain, striatum, and thalamus (**Figure 2**). VOI were between 10 mm3 (amygdala) and 0.3 cm3 (cortex). PET VOI statistics (kBq/cc) were generated for all these brain areas and standardized uptake values (SUV) were calculated [SUV = tissue activity concentration average (kBq/cc) × body weight (g)/ injected dose (kBq)] for semi-quantitative analysis. SUVs of ^18^F-FDG-PET scans were corrected for measured blood glucose levels [Glc = SUV × blood glucose level (mg/dl)]. SUVs of ^18^F-Florbetaben scans were further normalized to the SUV of the cerebellum VOI and the obtained ratios (SUVr) were used for further analysis.

### Morris Water Maze

The nocturnal animals were kept on an inverted 12 h/12 h dark/light cycle and tested during the dark period.

Male 7-month-old (*n* = 10) and 12-month-old (*n* = 10) 5XFAD and age-matched and sex-matched wild type mice (C57Bl/6J, *n* = 10–12) were tested in the Morris Water Maze (MWM) before PET/MRI imaging. MWM is a behavioral test for rodents, widely used to measure spatial reference memory (16, 17), and has been described previously (18, 19). In brief, the mice learn to use spatial cues in a circular pool filled with opaque water, in order to locate a hidden platform. The experiment began with 3 days of cued training during which the platform was marked with a triangular flag. Both the location of the platform and the position where mice were introduced into the pool changed between trials. Each mouse received four 60 s training trials per day with an average inter-trial interval of 15 min. Twenty-four hours after the last day of cued training, mice performed 5 days of acquisition training. For this part of testing, the flag was removed from the platform. In addition to the distal cues existing in the room, proximal visual cues were attached to the outside of the pool. The platform location remained stationary for each mouse throughout training. Trials were conducted as during the cued training phase. Twenty-four hours after the last acquisition trial, a probe test was performed. The probe trial was used to measure spatial memory by removing the hidden platform and introducing the mice into the water from a novel entry point. Mice were allowed to swim freely for 1 min while the swimming path was recorded. After the probe trial all mice were sacrificed. Swimming path, swimming speed, and quadrant preference where measured and analyzed using ANY-Maze video tracking software (Stoelting Co., Wood Dale, IL, USA).

### Immunohistochemistry on Paraffin Brain Sections

Mice were sacrificed at the age of 7 and 12 months, respectively. Brains were thoroughly removed, embedded in paraffin and cut into sections of 4 μm. Immunohistochemistry was carried out as described previously (20). The following antibodies were used: 2431-1 (1:500, pan-Abeta), GFAP (1:1000, rabbit, Synaptic Systems) and IBA-1 (1:1000, guinea pig, Synaptic Systems). Biotinylated anti-rabbit and anti-guinea pig antibodies (Jackson ImmunoResearch Laboratories, West Grove, PA, USA) were used as secondary antibodies. Staining was visualized by using the ABC method with a Vectastain Kit (Vector Laboratories, Burlingame, CA, USA) and diaminobenzidine as chromogen. Images were taken with an Olympus BX51 microscope equipped with a MoticamPro 282B digital camera. Microgliosis and astrogliosis were evaluated in the cortex area capturing serial images of 20x magnification on three sections per animal which were 30 μm afar from each other. Using ImageJ (V 1.51, NIH, Bethesda, MA, USA) the pictures were binarized to 16-bit black and white images and a fixed intensity threshold was applied defining the DAB staining. The percentage area covered by positive DAB staining was measured for each image (8, 15).

### Statistical Analysis

GraphPad Prism version 6 for Mac (GraphPad Software, San Diego, CA, USA) was used for all calculations. Differences between groups were tested with unpaired *t*-test or one-way analysis of variance (ANOVA) followed by Bonferroni multiple comparison as indicated. Data is given as mean +/- standard error of the mean (SEM). Significance levels are given as follows: ^*^p < 0.05; ^**^p < 0.01; ^***^p < 0.0001.

## Results

### Decreased Cerebral Glucose Metabolism in 5XFAD Mice

^18^F-FDG-PET was used to determine glucose metabolism in the brain of 7- and 12-month-old 5XFAD and wild type (WT) mice. ^18^F-FDG uptake was measured in the whole brain and in different brain regions using predefined VOIs and glucose corrected SUVs (SUVglc) were calculated. All tested mice showed ^18^F-FDG uptake in all cerebral areas and the cerebellum. Physiological extracranial distribution of ^18^F-FDG was visible with normal uptake in the Harderian glands, myocardium, brown adipose tissue, intestines, kidneys, and the urinary bladder.

**Figure 1:**
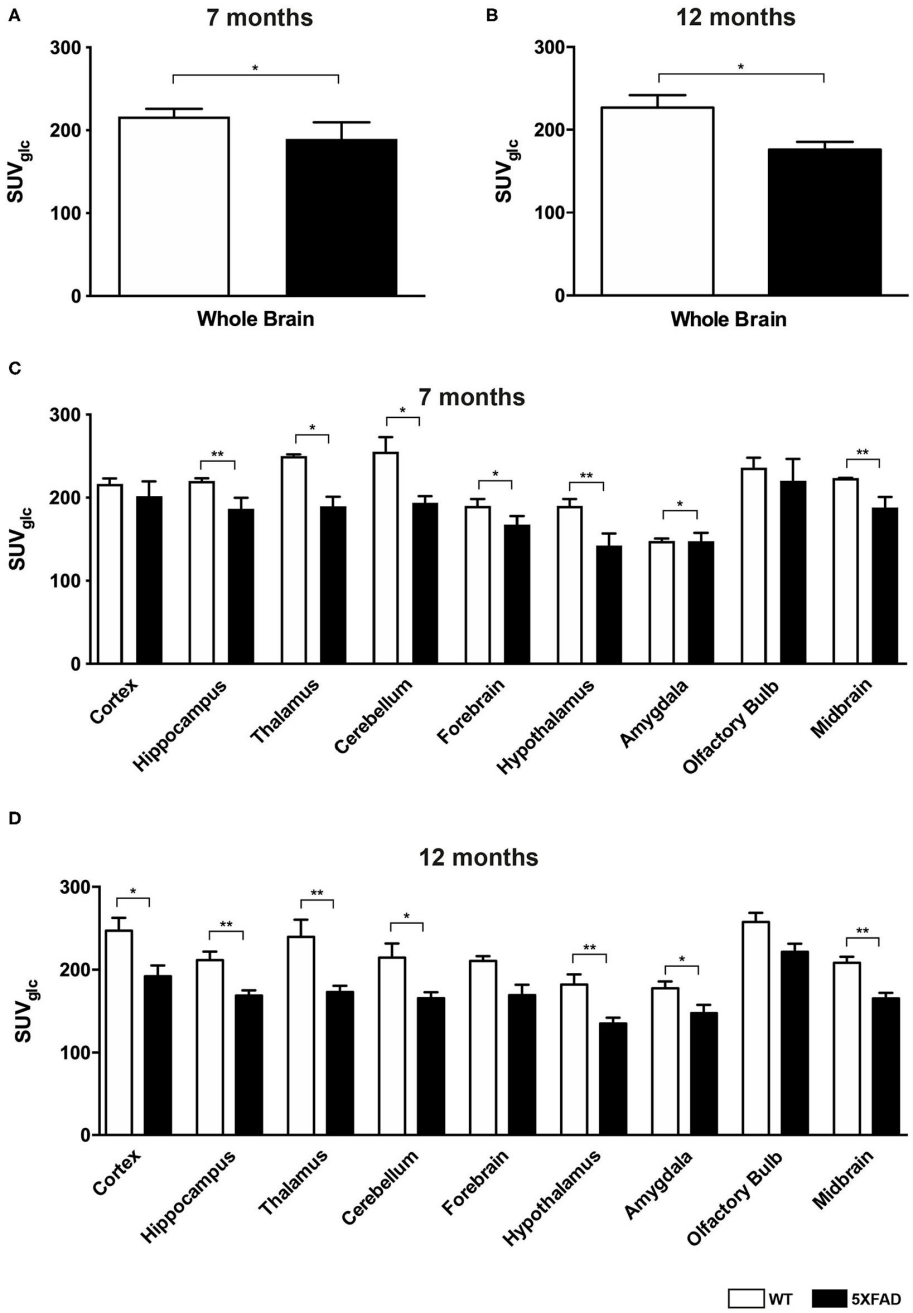
^18^F-FDG uptake in 7- and 12-month-old WT and 5XFAD mice. **(A)** 7-month-old 5XFAD mice showed a significantly lower SUVglc in the whole brain compared to age-matched WT mice. **(B)** 12-month-old 5XFAD mice showed a significantly lower SUVglc in the whole brain compared to age-matched WT mice. **(C)** 7-month-old 5XFAD mice showed a significantly lower SUVglc in all brain regions except for cortex and olfactory bulb compared to age-matched WT mice. **(D)** 12-month-old 5XFAD mice showed a significantly lower SUVglc in all brain regions compared to age-matched WT mice except for forebrain and olfactory bulb. Unpaired *t*-test; *p < 0.05; **p < 0.01; data presented as mean +/- SEM.

**Figure 2:**
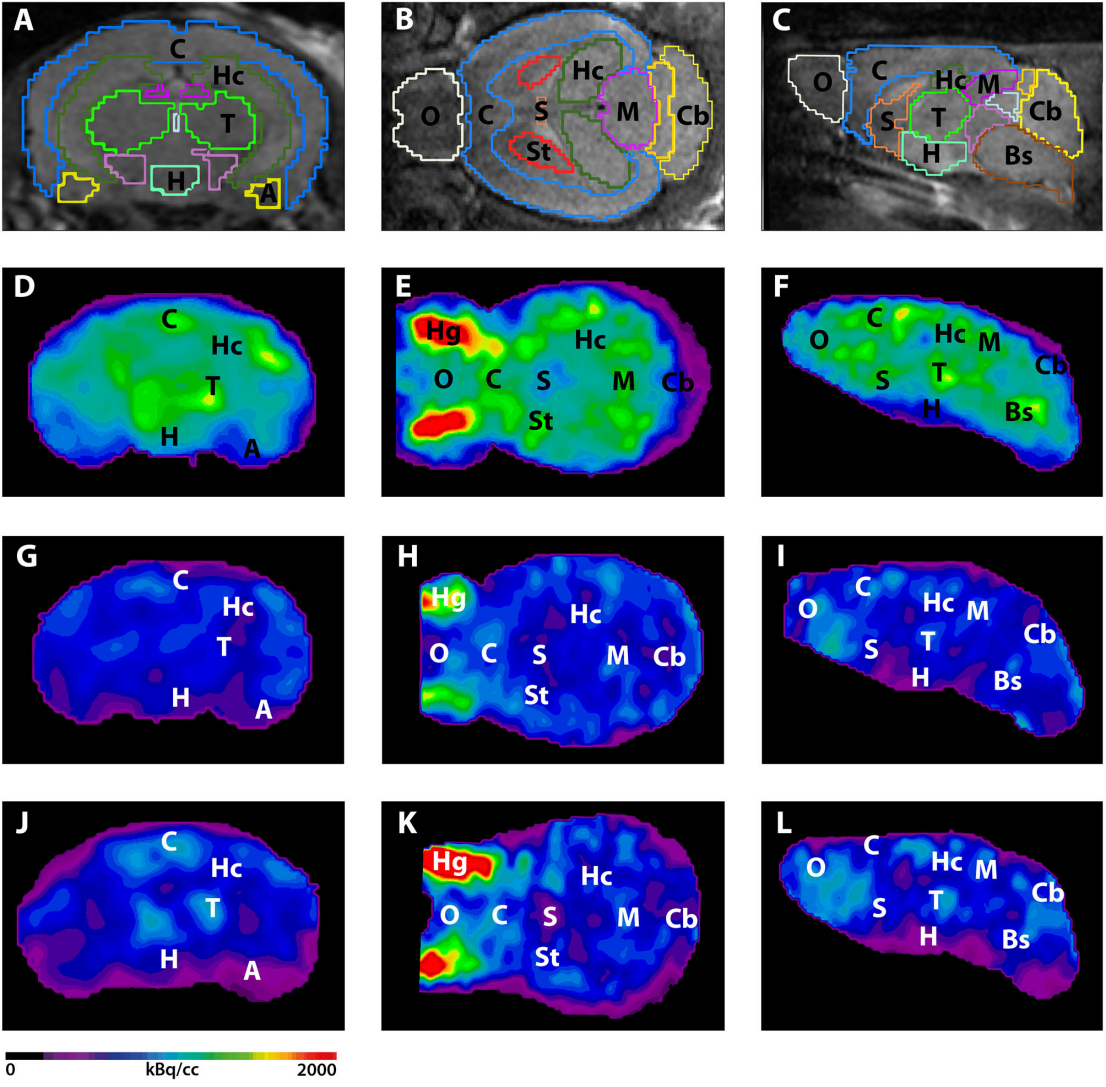
^18^F-FDG-PET images in coronal, transverse, and sagittal view. **(A–C)** MRI images with predefined brain regions. **(D–F)** ^18^F-FDG images of a representative 7-month-old WT mouse. **(G–I)** ^18^F-FDG-PET images of a 7-month-old 5XFAD mouse. 7-month-old 5XFAD mice showed distinctly lower FDG uptake compared to WT mice. **(J–L)** ^18^F-FDG images of a 12-month-old WT mouse. 12-month-old 5XFAD mice also showed significantly lower ^18^F-FDG uptake compared to age-matched WT mice. A, Amygdala; Bs, Brain Stem; C, Cortex; Cb, Cerebellum, H, Hypothalamus; Hc, Hippocampus; Hg, Harderian Glands; M, Midbrain; O, Olfactory Bulb; S, Septum/Basal Forebrain; St, Striatum; T, Thalamus.

Seven and 12-month-old WT mice did not show significant differences in whole brain uptake (*t*-test: *p* = 0.2672). 7-month-old 5XFAD mice showed lower SUVglc in the whole brain compared to age-matched WT mice (Figure 1A, *t*-test: *p* = 0.046). Differences between 7-month-old 5XFAD mice and WT mice were detected in all brain regions, except for cortex and olfactory bulb (Figure 1C, *t*-test: cortex: *p* = 0.0987; hippocampus: *p* = 0.0081; thalamus: *p* = 0.0341; cerebellum: *p* = 0.0313; forebrain: *p* = 0.0447; hypothalamus: *p* = 0.0044; amygdala: *p* = 0.018; olfactory bulb: *p* = 0.1527; midbrain: *p* = 0.0012). Twelve-month-old 5XFAD mice showed significantly lower SUVglc in whole brain uptake as well as in all brain regions compared to age-matched WT mice except for the olfactory bulb region (Figures 1B,D, *t*-test: whole brain: *p* = 0.0067; cortex: *p* = 0.0408; hippocampus: *p* = 0.0001; thalamus: *p* = 0.0103; cerebellum: *p* = 0.0004; forebrain: *p* = 0.0171; hypothalamus: *p* = 0.0038; amygdala: *p* = 0.0046; olfactory bulb: *p* = 0.0974; midbrain: *p* = 0.0001). Figure 2 shows exemplary ^18^F-FDG-PET results of a 7-month-old WT, a 7-month-old 5XFAD mouse and a 12-month-old 5XFAD mouse.

### Increased Amyloid Deposition in 5XFAD Mice

Amyloid PET using the tracer ^18^F-Florbetaben was used to determine amyloid plaque deposition in the brain of 7-and 12-month-old 5XFAD and WT mice. Quantitative analysis using nine VOIs was performed as described above. Cerebellum VOI was used as reference region and SUV ratios (SUVr) were calculated. Unspecific ^18^F-Florbetaben uptake was visible in all tested mice within brain, Harderian glands, intestines, and urinary bladder.

**Figure 3:**
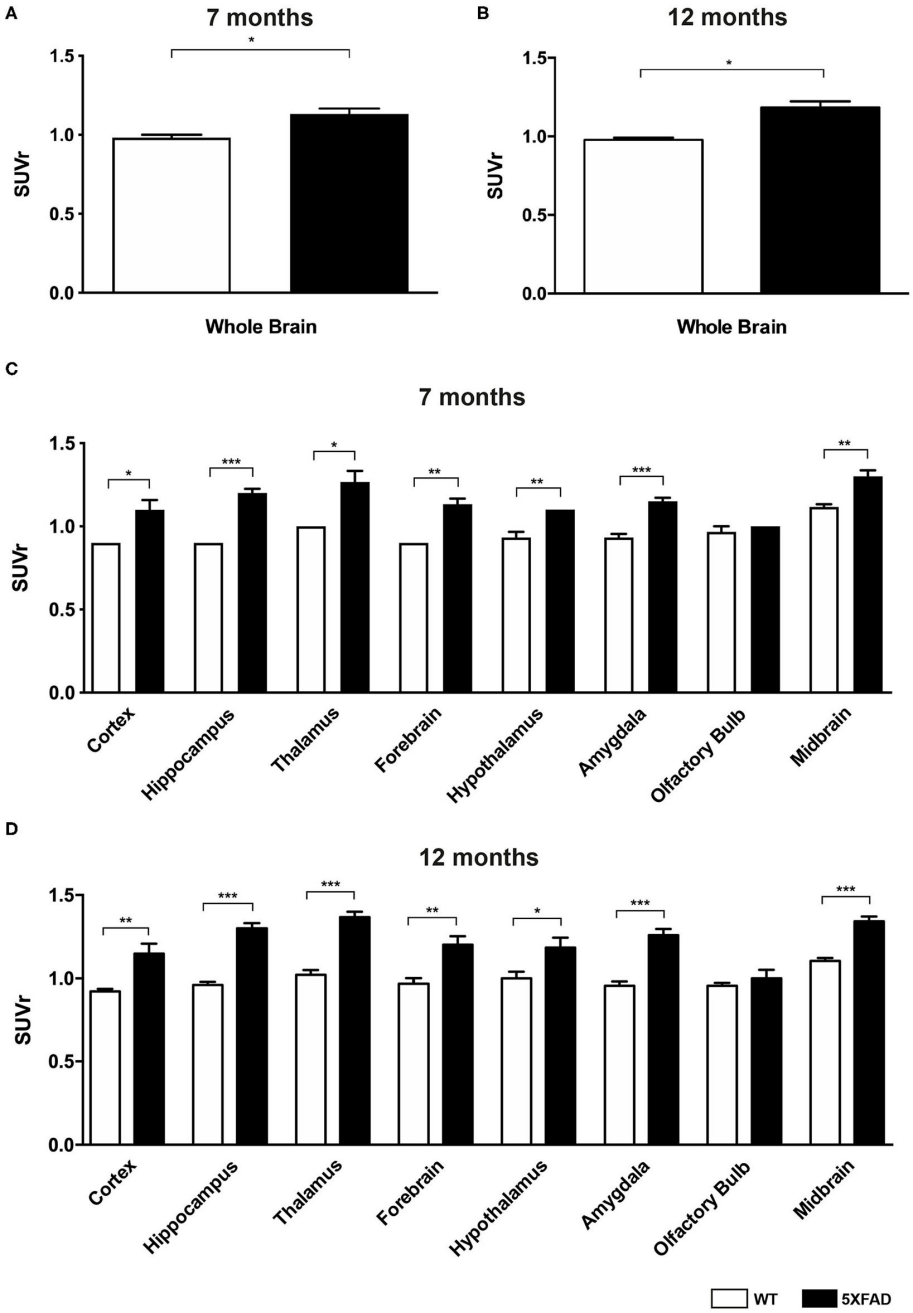
^18^F-Florbetaben uptake in 7-and 12-month-old WT and 5XFAD mice. **(A)** 7-month-old 5XFAD mice showed a significantly higher SUVr in the whole brain compared to age-matched WT mice. **(B)** 12-month-old 5XFAD mice showed significantly higher SUVr in the whole brain compared to age-matched WT mice. **(C)** 7-month-old 5XFAD mice showed a significantly higher SUVr in all tested brain regions compared to age-matched WT mice except for the olfactory bulb region. **(D)** Significant differences in 12-month-old mice were also detected in all analyzed brain regions except for the olfactory bulb. Unpaired *t*-test; *p < 0.05; **p < 0.01; ***p < 0.0001; data presented as mean +/- SEM.

**Figure 4:**
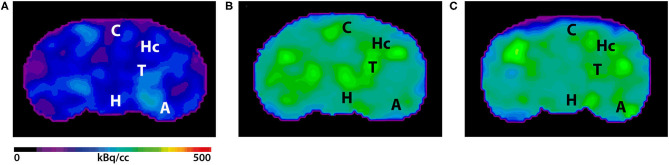
^18^F-Florbetaben-PET images in coronal view. **(A)** ^18^F-Florbetaben image of a 7-month-old WT mouse. **(B)** Cerebral ^18^F-Florbetaben uptake was higher in 7-month-old 5XFAD mice as well as in 12-month-old 5XFAD mice **(C)**. A, Amygdala; C, Cortex; H, Hypothalamus; Hc, Hippocampus; T, Thalamus.

Seven- and 12-month-old WT mice did not show significant differences in whole brain uptake (*t*-test: *p* = 0.6263). SUVr in the whole brain region of 7-month-old 5XFAD mice was significantly increased compared to age-matched WT mice (Figure 3A, *t*-test: *p* = 0.0241). Increased ^18^F-Florbetaben uptake was detected in all tested brain regions except for the olfactory bulb (Figure 3C, *t*-test: cortex: *p* = 0.0257; hippocampus: *p* < 0.0001; thalamus: *p* = 0.016; forebrain: *p* = 0.0022; hypothalamus: *p* = 0.0075; amygdala: *p* < 0.0001; olfactory bulb: *p* = 0.3739; midbrain: *p* = 0.001). Seven-and 12-month-old 5XFAD mice did not show significant differences in whole brain uptake (*t*-test: *p* = 0.4512;). Twelve-month-old 5XFAD mice showed increased ^18^F-Florbetaben uptake in the whole brain (Figure 3B, *t*-test: *p* = 0.0106) and all tested brain regions except for the olfactory bulb compared to age-matched WT mice (Figure 3D, *t*-test: cortex: *p* = 0.0004; hippocampus: *p* < 0.0001; thalamus: *p* < 0.0001; forebrain: *p* = 0.0015; hypothalamus: *p* = 0.021; amygdala: *p* < 0.0001; olfactory bulb: *p* = 0.359; midbrain: *p* < 0.0001). Figure 4 shows examples of ^18^F-Florbetaben-PET results of a 7-month-old WT as well as a 7-month-old 5XFAD and a 12-month-old 5XFAD mouse.

### Plaque Load and Gliosis in 5XFAD Mice

**Figure 5:**
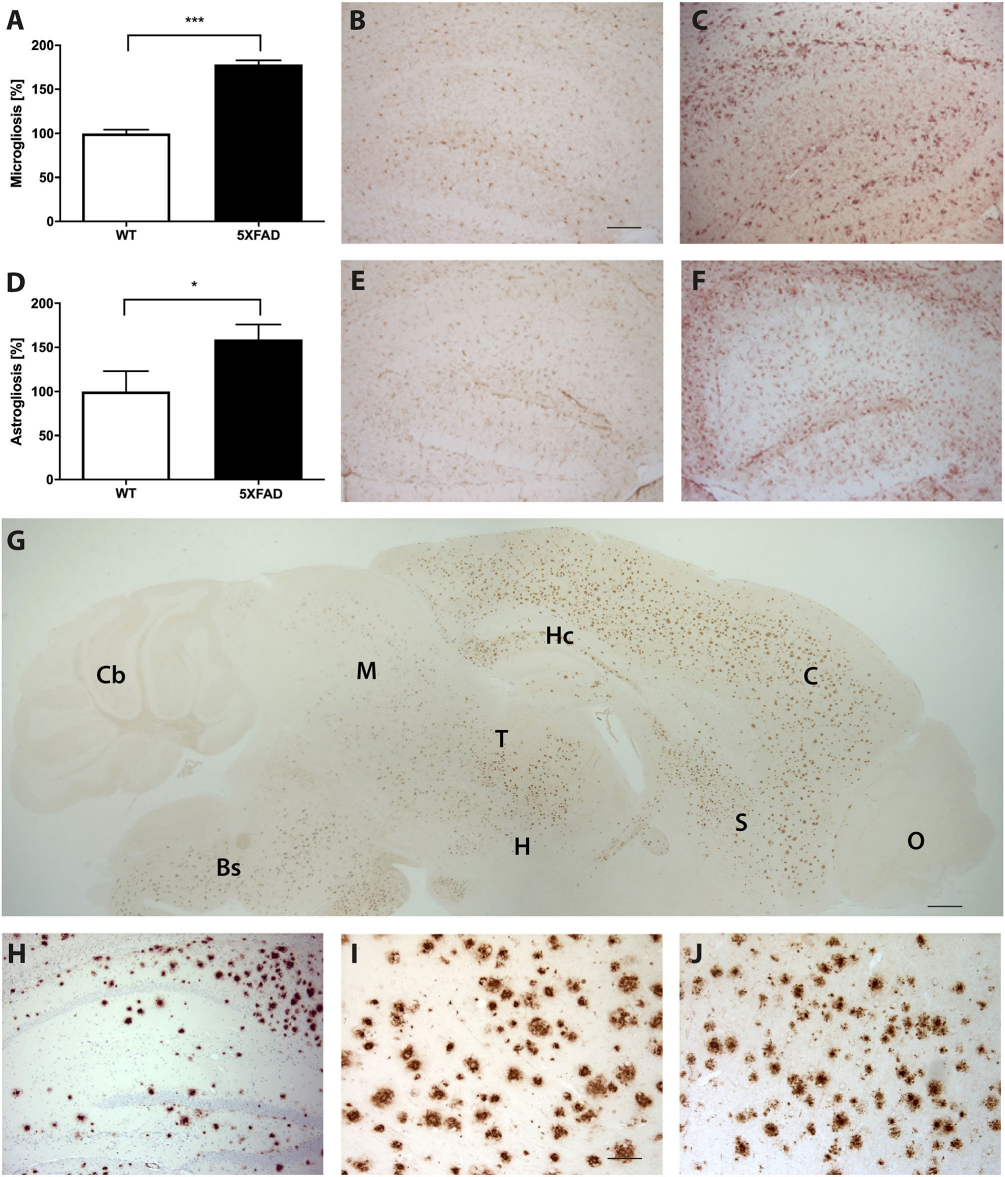
Increased astrogliosis, microgliosis, and Aβ-expression in 5XFAD mice. **(A)** Significantly more reactive microglia activity in the brain of 7-month-old 5XFAD mice compared to age-matched WT controls. Examples of hippocampal sections of **(B)** WT and **(C)** 5XFAD mice. **(D)** 5XFAD mice displayed significantly more astroglia reactivity than WT controls. Examples of hippocampal sections of **(E)** WT and **(F)** 5XFAD. **(G)** Aβ-expression in 5XFAD mice shows plaque pathology in various brain regions except of olfactory bulb and cerebellum. Bs, Brain Stem; C, Cortex; Cb, Cerebellum, H, Hypothalamus; Hc, Hippocampus; M, Midbrain; O, Olfactory Bulb; S, Septum/Basal Forebrain; T, Thalamus. **(H–J)** Examples of plaque pathology in the **(G)** hippocampus, **(H)** cortex, and **(I)** thalamus of 5XFAD mice. Scale bars in **(B)** for **(C,E,F,H)** = 200 μm; Scale bar in G = 500 μm; Scale bar in **(I)** for J = 50 μm. Unpaired *t*-test; ***p < 0.0001; *p < 0.05; data presented as mean ± SEM.

Brain sections of 5XFAD mice stained with an anti-Aβ antibody showed a severe plaque load in 7-month-old 5XFAD mice (Figures 5G–J). Amyloid plaques were detected throughout the brain but not in the olfactory bulb or cerebellum. Immunohistochemical IBA1 staining revealed 78% more reactive microglia in 5XFAD mice compared to WT animals (Figures 5A–C, *t*-test: *p* < 0.001). In addition, 5XFAD mice displayed a significantly increased astroglia activity (Figures 5D–F, *t*-test: *p* = 0.0393).

### Memory Deficits in 5XFAD Mice in the Morris Water Maze

**Figure 6:**
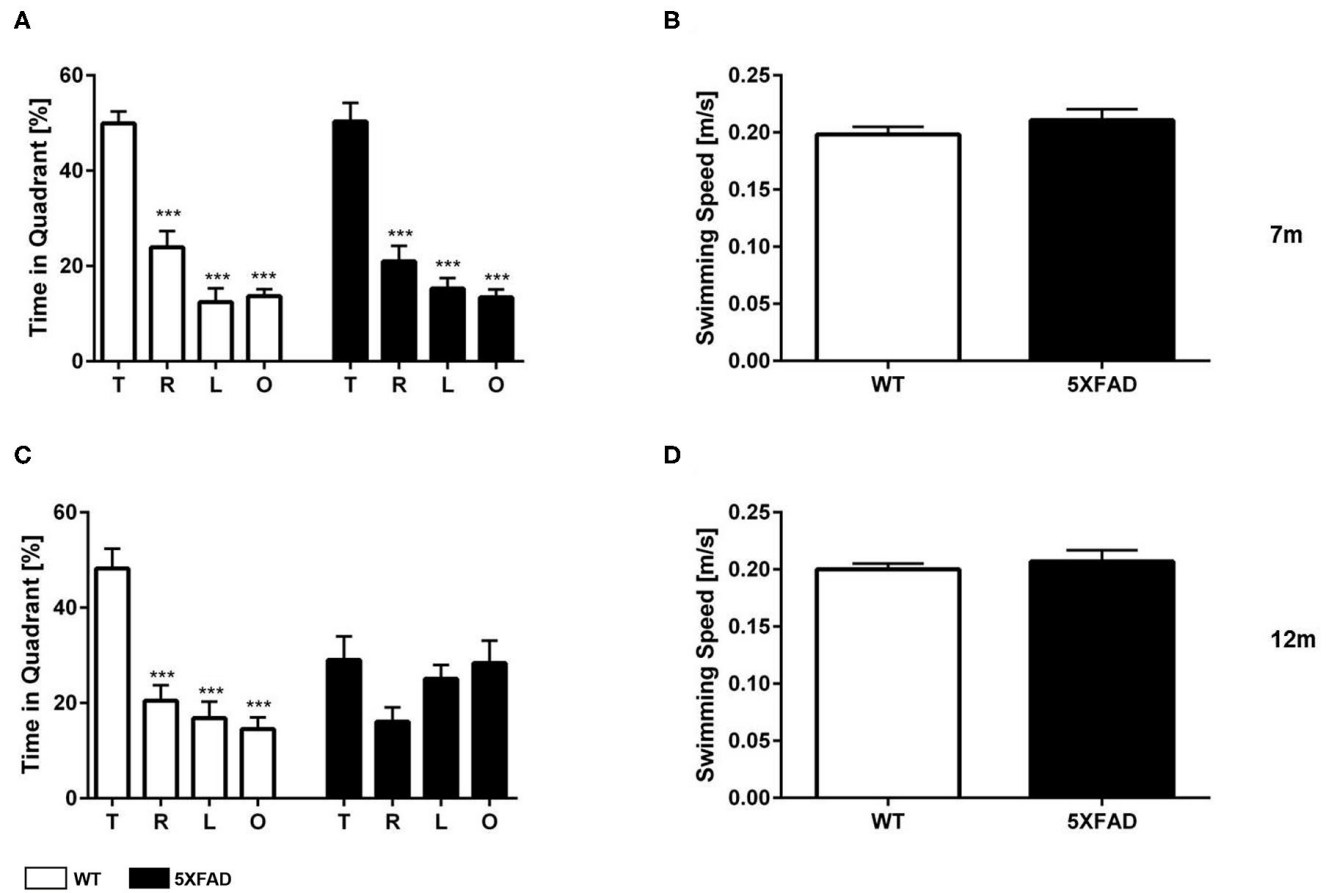
Spatial reference memory deficits in 5XFAD mice in the Morris water maze. **(A)** 7-month-old WT and 5XFAD mice showed no memory deficits in the probe trial. **(C)** At 12 months of age male 5XFAD showed no quadrant preference revealing an impaired spatial reference memory. In contrast, 12-month-old WT animals displayed an intact reference memory. **(B,D)** Swimming speed was not altered at any of the tested time points. **(A,C)** One-way ANOVA followed by Bonferroni multiple comparisons. **(B,D)** Unpaired *t*-test; ***p < 0.0001; *T*, target quadrant; *L*, left quadrant; *R*, right quadrant; *O*, opposite quadrant; data presented as mean +/- SEM.

The probe trial of the MWM was used to test spatial reference memory in 7- and 12-month-old 5XFAD and WT mice. WT mice, independent of age, and 7-month-old 5XFAD mice showed a significant preference for the target quadrant, as indicated by the percentage time spent the four quadrants of the pool (Figures 6A,C *one-way ANOVA*: *p* < *0.001 target vs. all other quadrants*).

In contrast, 12-month-old 5FAD mice displayed no significant preference for the target quadrant (Figure 6C, *one-way ANOVA*). The lack of preference for any quadrant demonstrates that 12-month-old 5XFAD mice have a robust deficit in spatial reference memory. Swimming speed was not altered in 5XFAD mice at any time-point tested (Figures 6B,D, *t*-test: 7 m: *p* = 0.3561, 12 m: *p* = 0.4217).

## Discussion

Nuclear medicine imaging of the human AD brain has advanced over the last decades and the imaging biomarkers ^18^F-FDG- and amyloid-PET became clinically well-established in the diagnostic workup of AD patients (21). While ^18^F-FDG-PET is able to assess synaptic dysfunctions, amyloid-PET detects cerebral amyloid plaque deposition (22–25). Both, synaptic dysfunction and plaque deposition, are described as early changes in the development of AD (26, 27). Therefore, these pathologies are interesting targets for imaging the disease in an early stage as well as for the evaluation of disease progression and therapeutic efficacies.

As there is still no cure for AD, preclinical studies on new therapeutic strategies are essential and ^18^F-FDG- and amyloid-PET could serve as therapeutic readouts in these studies. With the availability of small animal PET scanners, PET imaging is a perfect cross-species tool that allows the measurement of the same molecular processes in animals and humans. Utilization of PET tracers allows longitudinal examination of various pathological changes and additionally increases the quality of preclinical studies evaluating therapeutic efficacy of novel therapeutics by monitoring molecular changes *in vivo*.

The 5XFAD mouse model is a commonly used familial AD model and often utilized in preclinical studies evaluating possible disease modifying drugs for AD. The 5XFAD model with five AD-linked mutations is a model of brain amyloidosis that exhibits AD-like phenotypes (28–31). 5XFAD mice show early and massive plaque formation, intraneuronal Aβ aggregation and neuron loss in the subiculum and neocortical layer five as well as memory deficits (6, 9, 32). Due to its key features, the 5XFAD model is in theory a suitable model for ^18^F-FDG-and amyloid-PET studies as it develops a severe plaque pathology as well as synaptic dysfunctions (9, 33). However, only a few studies using PET in the 5XFAD model have been performed so far and the suitability of the method in this mouse model remains unclear, especially the use of ^18^F-FDG-PET (34).

Available studies on ^18^F-FDG-PET in 5XFAD mice showed contradictory findings. ^18^F-FDG-PET is used as a tool for the assessment of neuronal glucose metabolism which is mainly determined by synaptic activity. AD patients show a decrease of neuronal activity especially in the temporal and parietal cortex. Patients in early stages of AD usually show a reduced glucose metabolism in ^18^F-FDG-PET within the posterior cingulate cortex. During the progress of the disease lower ^18^F-FDG uptake can also be measured in the posterior temporal and parietal cortex and eventually in the frontal lobe (35–39).

In 5XFAD mice, some studies, including our results presented here, show comparable results to typical ^18^F-FDG-PET findings in AD patients. In the current study, which is the first preclinical study utilizing PET/MRI in 5XFAD mice, 7- and 12-month-old 5XFAD mice showed distinct reduced ^18^F-FDG uptake in the whole brain as well as in several brain regions. Macdonald et al. (12) described a significant decrease of ^18^F-FDG uptake in 13-month-old 5XFAD mice, while younger animals did not show differences in the whole brain SUV compared to WT. Another study by Xiao et al. (40) reported lower ^18^F-FDG uptake in the cerebral cortex, hippocampus and olfactory bulb of 6-month-old 5XFAD mice. Hypometabolism within the olfactory bulb was detected as early as 3 months of age (40). Our results could not confirm changes within the olfactory bulb as described by Xiao et al. (40). Only two other studies on 18F-FDG-PET in AD mouse models analyzed the olfactory bulb regions so far. Both studies did not show significant differences between transgenic APP/PS1-21 or Tg2576 and WT mice (41, 42). Furthermore, in our study amyloid pathology was not detected in the olfactory bulb with 18F-Florbetaben or immunohistochemistry. It can be assumed that abundant plaque pathology leads to changes in brain metabolism and therefore the lack of abundant plaque pathology might explain missing changes in glucose metabolism.

In contrast to our results, Rojas et al. (11) showed an increased ^18^F-FDG uptake relative to the cerebellum in 11-month-old 5XFAD mice. A common explanation of increased cerebral glucose metabolism has been the presence of activated inflammatory cells around amyloid plaques (11, 43). Amyloid plaques are pro-inflammatory agents and an increased activation of microglia and astroglia could therefore lead to an increased cerebral FDG uptake. However, we could demonstrate a cerebral hypometabolism in 5XFAD mice despite an increased astrogliosis and microgliosis. Similar to our results, significant cerebral hypometabolism was detected by ^18^F-FDG-PET in several other AD models with known increased gliosis including Tg4-42, Tg2576, TASTPM, and APPS1-21 (15, 41–43). In addition, AD patients also display a cerebral hypometabolism and increased gliosis (44–46). Therefore, increased gliosis does not seem to explain those findings.

As previously discussed (34), discrepancies between animal studies might primarily be explained by different image acquisition and normalization methods. In several studies on ^18^F-FDG-PET in transgenic mouse models of AD, including the study by Rojas et al. (11), the cerebellum was used for normalization of cortical FDG uptake. In AD patients the cerebellum is often used as reference region as glucose metabolism is relatively preserved in the cerebellum during disease progression. However, AD pathologies in transgenic mice seem to also influence FDG uptake in the cerebellum (47). Next to the findings of Rojas et al. (11), studies that normalized cerebral FDG uptake to the cerebellum also showed higher ^18^F-FDG uptake in APP/PS1 and APP/PS2 mice (11, 48–50). This might explain findings by Rojas et al. (11) in 5XFAD mice.

In addition, blood glucose levels highly influence ^18^F-FDG uptake in the brain. Even with standardized pre-imaging protocols blood glucose levels can vary between individual animals due to stress, body temperature or fasting durations (51). Furthermore, blood glucose levels of transgenic mice are known to be lower after a fasting period compared to WT mice (41, 47). As ^18^F-FDG uptake shows an inverse relationship to blood glucose levels, normalization of ^18^F-FDG uptake to blood glucose should be considered for creating reliable and reproducible data.

Next to the assessment of neuronal dysfunctions via ^18^F-FDG-PET, PET neuroimaging tools also allow the evaluation of amyloid burden *in vivo*. ^18^F-Florbetaben, ^18^F-Florbetapir, ^18^F-Flutemetamol and ^11^C-labeled Pittsburgh Compound-B (^11^C-PIB) are widely used PET tracers that visualize Aβ-plaque deposits *in vivo* (22, 23, 25, 52, 53). Despite some initial negative results and a lower affinity for fibrillar amyloid-beta (54–56), amyloid imaging has been successfully back-translated into several transgenic mouse models of AD (57, 58). In addition, it has been demonstrated that amyloid tracers are sensitive enough to detect treatment-related plaque load reduction *in vivo* in a number of amyloid mouse models (59–61).

The presence of an extensive plaque pathology in 5XFAD mice has been well-described (6, 8, 62). Extracellular amyloid plaques were detected starting at 2 months of age aggravating over time (6, 62).

However, only a few studies on amyloid-PET in 5XFAD mice have been published so far. Rojas et al. (11) described a higher cerebral uptake of ^11^C-PIB and ^18^F-Florbetapir in the brain of 11-month-old 5XFAD mice compared to WT animals (11). In addition, Oh et al. (13) reported elevated uptake of the amyloid tracer ^18^F-FC119S in the hippocampus, thalamus, and cortex of 5.5-month-old 5XFAD mice. Our results with ^18^F-Florbetaben are well in line with these findings showing an increased ^18^F-Florbetaben tracer uptake in 7- and 12-month-old 5XFAD mice.

In this study we could show that both imaging biomarkers, ^18^F-FDG- and ^18^F-Florbetaben-PET, are useful tools for the *in vivo* detection of cerebral AD pathologies in 5XFAD mice even before the onset of severe memory deficits. The 5XFAD mouse model of AD therefore is a suitable model for preclinical PET studies showing comparable changes to AD patients with the clinically established biomarkers ^18^F-FDG and ^18^F-Florbetaben. Therefore, PET imaging can be utilized as a readout for therapeutic effects *in vivo* in future longitudinal therapy studies using the 5XFAD mouse model of AD.

Limitations of the study include possible partial volume effect, which can especially affect analysis of smaller VOIs. VOIs in our study were mapped on PET images with the help of an MRI-based mouse brain atlas. Volume of the smallest VOI (amygdala) was 10 mm3; right above a suggested threshold to avoid partial volume effect in small animal scanners of 9 mm3 (63, 64). The manufacturer describes spatial resolution of the used PET/MRI scanner up to 0.7 mm [MEdiso Website; (65)]. Therefore, partial volume effect might have been avoided. However, influence of partial volume effect on analysis of smaller VOIs cannot be absolutely excluded.

## Conclusion

Our results support the 5XFAD mouse model as a reliable model for AD research. *In vivo* PET imaging with ^18^F-FDG and ^18^F-Florbetaben displays a suitable tool for *in vivo* monitoring of AD pathologies and therapeutic efficacy in 5XFAD mice.

## Data Availability Statement

The raw data supporting the conclusions of this article will be made available by the authors, without undue reservation.

## Ethics Statement

The animal study was reviewed and approved by Niedersächsisches Landesamt für Verbraucherschutz und Lebensmittelsicherheit, Röverskamp 5, 26203 Oldenburg, Germany and Landesamt für Gesundheit und Soziales LAGeSo Darwinstr. 15, 10589 Berlin, Germany.

## Author Contributions

TF and CI performed experiments, analyzed data, and contributed to writing the manuscript. NB and WB performed experiments. TB participated in the discussion of the results. CB and YB designed the project, performed experiments, analyzed data, and wrote the manuscript. All authors contributed to revising the manuscript and approved the final version. All authors contributed to the article and approved the submitted version.

## Conflict of Interest

The authors declare that the research was conducted in the absence of any commercial or financial relationships that could be construed as a potential conflict of interest.
